# Electronic Descriptors for Supervised Spectroscopic Predictions

**DOI:** 10.1021/acs.jctc.2c01039

**Published:** 2023-03-06

**Authors:** Carlos Manuel de Armas-Morejón, Luis A. Montero-Cabrera, Angel Rubio, Joaquim Jornet-Somoza

**Affiliations:** † Nano-Bio Spectroscopy Group, Departamento de Polímeros y Materiales Avanzados: Fisica, Química y Tecnología, Universidad del País Vasco UPV/EHU, 20018 San Sebastián, Spain; ‡ Laboratorio de Química Computacional y Teórica, Facultad de Química, Universidad de La Habana, 10400 La Habana, Cuba; ¶ Donostia International Physics Center, Manuel Lardizabal Ibilbidea, 4, 20018 Donostia, Spain; § Theory Department, Max Planck Institute for the Structure and Dynamics of Matter and Center for Free-Electron Laser Science, Luruper Chaussee 149, 22761 Hamburg, Germany

## Abstract

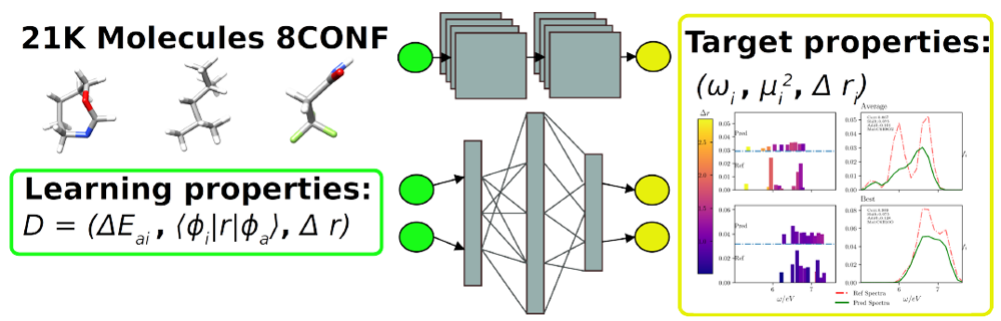

Spectroscopic properties of molecules hold great importance for the description of the molecular response under the effect of UV/vis electromagnetic radiation. Computationally expensive *ab initio* (e.g., MultiConfigurational SCF, Coupled Cluster) or TDDFT methods are commonly used by the quantum chemistry community to compute these properties. In this work, we propose a (supervised) Machine Learning approach to model the absorption spectra of organic molecules. Several supervised ML methods have been tested such as Kernel Ridge Regression (KRR), Multiperceptron Neural Networs (MLP), and Convolutional Neural Networks. [Ramakrishnan et al. J. Chem. Phys.2015, 143, 084111.2632882210.1063/1.4928757Ghosh et al. Adv. Sci.2019, 6, 1801367.] The use of only geometrical-atomic number descriptors (e.g., Coulomb Matrix) proved to be insufficient for an accurate training. [Ramakrishnan et al. J. Chem. Phys.2015, 143, 084111.2632882210.1063/1.4928757] Inspired by the TDDFT theory, we propose to use a set of electronic descriptors obtained from low-cost DFT methods: orbital energy differences (Δϵ_*ia*_ = ϵ_*a*_ – ϵ_*i*_), transition dipole moment between occupied and unoccupied Kohn–Sham orbitals (⟨ϕ_*i*_|*r*|ϕ_*a*_⟩), and when relevant, charge-transfer character of monoexcitations (*R*_*ia*_). We demonstrate that with these electronic descriptors and the use of Neural Networks we can predict not only a density of excited states but also get a very good estimation of the absorption spectrum and charge-transfer character of the electronic excited states, reaching results close to chemical accuracy (∼2 kcal/mol or ∼0.1 eV).

## 1 Introduction

The absorption spectra hold great importance for discovering photoelectric features in chemistry and materials science. The design of new photosensitive devices and materials for the energy industry as well as healthcare has become a hot topic in the last decades. A fast and accurate method that enables discrimination between hundreds or thousands of candidates becomes crucial to speed up new material discoveries with desired spectroscopic properties. The increase of the experimental and *ab initio* theoretical databases^1,3^ on materials pushed forward a new way for their design, but usually they do not incorporate all the required spectroscopic information. Then, researchers rely on quantum mechanics techniques, usually Time-Dependent Density Functional Theory (TDDFT)^4,5^ or multiconfigurational wave function methods,^6^ for a rather confident prediction of properties and characterization. However, these types of calculations are usually complex to perform and to understand for nontrained researchers, particularly when trying to get reliable predictions of absorption spectra from an initial selection within several candidates.

Recently, Machine Learning (ML) algorithms have attracted the interest of the research community because the plausible results obtained predicted materials properties with good accuracy.^1,7^ ML algorithms have been used, for example, for property classifications and group discovery,^9,10^ as well as ground-state material and molecular property predictions.^7,11−13^ It could also be very useful for understanding the nature of many molecular properties. The case of electronic excitations is able to be understood beyond the usual and very rough orbital descriptions, as they used to be based on rather approximate one-electron wave functions.^14^ In addition, the so-called “inverse molecular design” could be aided if similarities among ML descriptors are appropriately used for such purposes.^15^

Profound research on several ML methods to be chosen, such as supervised or unsupervised models, kernel regression methods, or neural networks, etc., is required for each type of target property. Moreover, the choice of the appropriate molecular descriptors has to be made carefully in order to fulfill some desired criteria: 1) simplicity: must be easy to produce, 2) representability: must contain the required information correlated to the target property, and 3) specificity: must be unique enough to distinguish between different molecules.^16^ Several descriptors have been proposed in the literature with different levels of applications.^9,17−47^ Ouyang et al.^9^ propose also the SISSO method for constructing these molecular or material descriptors based on algebraic combinations of atomic properties.

Several attempts have been made to predict theoretical spectroscopic properties for molecules^1,13^ and materials.^12,16,17,48^ The seminal work done by Ramakrishnan et al.^1^ proposed a kernel ridge regression model that can predict the first excited state with good results. Besides, they proposed a method called Δ*ML* for the estimation of the shift between two databases obtained using different Exchange-Correlation (XC) functionals. In that work, the authors used the so-called Coulomb Matrix^7^ as a geometrical descriptor related to atomic numbers of vertex elements, which has gained notoriety because of its low computational requirements and its good performance for predicting molecular properties.^1,2^ However, it proved to be insufficient for the proper prediction of the transition probability.^1^ In a recent work, Westermayr et al. found a machine learning model based on the use of a complex Neural Network that using many conformers of the same molecule as a training set can be used to accurately predict its absorption spectra.^49^

In this work, we propose for the first time the use of some calculated electronic properties in order to well characterize the spectroscopic fingerprint of small molecules. By using a simple Convolutional Neural Network model trained by low-cost theoretical electronic calculations obtained from a 21k molecular database, we can predict excitation energies together with their corresponding charge-transfer character and oscillator strength. The results presented in this paper are obtained by employing electronic descriptors from ground-state DFT calculations using a simple LDA XC-functional to predict the absorption spectra at a TDDFT level using the PBE0 hybrid XC-functional. The validity of the selected model is contrasted with different Neural Network schemes, and the limitations are described on the basis of obtained results. Hence, the resulting trained Neural Network can be used to predict one or a large number of molecules with minimal computational cost.

### 1.1 Molecular Database and Descriptor Selection

In this work, we take a subset of the *GDB-8* molecular database also used by Ramakrishnan et al.^1,3^ It consists of 21k small organic molecules with relaxed geometries computed at the DFT level by using Gaussian09 with the B3LYP/6-31(2df,p) functional.^44^ The selected molecules contain up to 8 carbon (C), oxygen (O), nitrogen (N), and/or fluor (F) atoms, being the number of hydrogen atoms required to make neutral the molecular charges. Hereon, we will refer to it as the 8CONF database.

Although the Sorted Coulomb Matrix and its variants have previously shown good results for the prediction of excitation energy levels and density of states,^1^ the use of only geometrical molecular descriptors proved to be insufficient for the correct prediction of transition moments and oscillator strengths.

For that reason, we propose to use electronic molecular descriptors from low-cost theoretical calculations (ground-state’s LDA) to predict accurate spectroscopic properties computed at the TDDFT level with a hybrid exchange-correlation functional (PBE0). We use the Octopus^51^ code to compute all electronic descriptors. The current version of the code includes all required features and utilities to obtain them.^52^ No further structural relaxation with the PBE0 functional for the training set was carried out in order to get predictions of spectroscopic information based on pure electronic descriptors.

The choice of the electronic descriptor has been made regarding the linear-response time-dependent DFT formulation (**LR-TDDFT**).^4,5^ This approach aims to solve the time-dependent Shrödinger equation
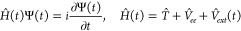
1where  is the system Hamiltonian composed by a kinetic part , an electron–electron potential component , and the all-other types of interaction contained in the time-dependent external potential term . Usually, the latter contains the nuclei-electron and external field interaction.

In LR-TDDFT, the time-dependent evolution of the noninteracting system under an external field is described by the noninteracting *density–density* response function χ_*s*_(***r***, ***r***′, ω)
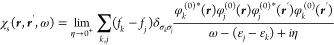
2where φ_*j*_ stands for Kohn–Sham (KS) orbitals, ϵ_*j*_ and *f*_*j*_ stand for their corresponding energies and occupations, respectively, and  is a Kronecker delta for orbital *j* and *k* spin functions. Finally, ω is the frequency of the perturbing external field, and η is a positive infinitesimal.

This function has poles on the excitation energy of the KS system. In order to obtain the excitation energies of the full interacting system, we have to solve the Dyson-like equation. Casida et al. proposed a matrix formulation to solve this equation, and he obtained the well-known Casida’s equation.^53^ By solving this equation, the excitation energy levels and oscillator strengths are obtained as a combination of the biorbital function  where subindices *a* and *i* correspond to unoccupied and occupied states, respectively.

In the present work, we use the excitation energies and oscillator strengths of 15k molecules computed using Casida’s equations at the PBE0 functional level in the ground state, both to train our ML model and as targets to validate it.

Let us return to the LR-TDDFT formulation in order to define the descriptors we will use. The time evolution of the polarizability function is defined in LR-TDDFT as the dipole–dipole response function, which in the space of frequencies takes the form
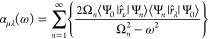
3where Ψ_0_ is the actual ground-state wave function of the interacting system, Ψ_*n*_ is the *n*-th excited-state wave function, Ω_*n*_ is the excitation energy of the *n*-th excited state, μ and λ are directions in the space, and  is the dipole operator in the ν direction.

This function has also poles in the excitation energies of the system, and the corresponding oscillation strengths are proportional to the numerator. Therefore, it is also used to compute the absorption spectra of molecular systems.

Based on eq 3 and the use of biorbital functions representing monoelectronic excitations, we propose to use the following electronic descriptors:

1. Orbital energy difference: Δϵ_*ia*_ = ϵ_*a*_ – ϵ_*i*_.
2. Kohn–Sham transition moment:

Nevertheless, the only use of these two properties does not fulfill the desired criteria of specificity described above. The calculated oscillator strengths depend on orbital overlapping between unoccupied and occupied states and are hence proportional to transition dipole moments. Besides, a work of Guido et al.^21^ proposes an easy way to evaluate the charge-transfer character of an excitation by defining a new index, Δ***r***
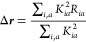
4
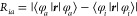
5
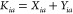
6where the intervening excitation *X*_*ia*_ and de-excitation *Y*_*ia*_ coefficients of the non-Hermitean solution correspond to the TD formalism.

We would like to remark that, although *R*_*ia*_ vanishes for centrosymmetric molecules and, therefore, does not introduce information in such cases, it remains relevant for all others. A zero value of one of the descriptors for them means that they remain only defined by those evaluated, keeping the full model of descriptors for the rest of the database.

**Figure 1:**
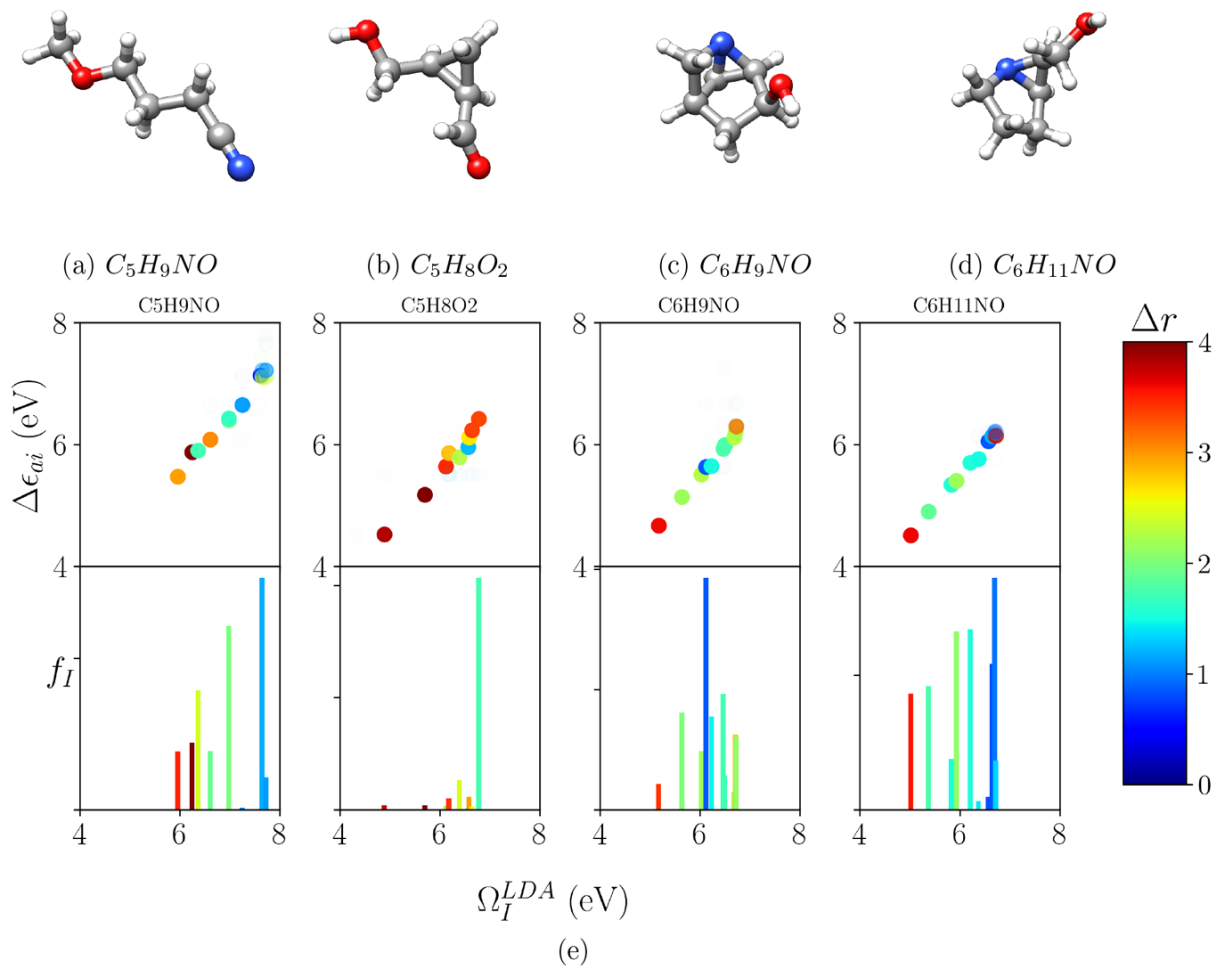
(a-d) Examples of four selected molecules from the 8CONF database. C, H, N, and O atoms are represented in gray, white, blue, and red, respectively. (e) Correlation between orbital energy differences and LR-TDDFT calculated excitation energies  (first row from top to bottom). The second row shows the calculated discrete absorption spectra for those molecules corresponding to such excitation energies. Transparency is proportional to Casida’s coefficient, and color is proportional to the charge-transfer character.

Following that index and knowing that only symmetrically similar monoexcitation contributes to the real excited state (Figure 1), we decided to also include the charge-transfer character of KS monoexcitations as a descriptor, as well as the TDDFT charge-transfer index of the excited state as a target property to predict.

Consequently, in this work, we propose the use of the combination of three electronic descriptors, namely (i) Δϵ_*ia*_, (ii) , and (iii) *R*_*ia*_, computed at the ground-state LDA XC-functional level and LCAO (LDA), for the 20 lowest-lying monoelectronic transitions in order to predict three spectroscopic properties for the first ten excitations: a) excitation energy (Ω_*I*_), b) oscillator strength (*f*_*I*_), and c) charge-transfer character (Δ***r***) at the PBE0 accuracy level.

### 1.2 Supervised Machine Learning Models

In this work, we use Neural Networks (NNs) because of their recognized versatility to find hidden correlations among several properties. We explore different NN models such as the Multi-Layer Perceptron (MLP) and the Convolution Neural Network (CNN). Each model depends on a group of internal variables, known as hyperparameters, such as the number of hidden layers, the number of neurons per layer, the number of learning iterations (also known as *epochs*), the activation functions, and many others. Some of these variables need to be optimized to fine-tune the NN.

Hyperparameter optimizations hold great importance for the correct behavior of the NN. Their values can be empirically selected, but the best combination can be only achieved by a systematic search. Then, we applied a Bayesian Optimization as implemented in *scikit-learn*(54) to find the optimal values for the *number of hidden layers* and the *number of epochs*.

Another important issue is how to feed the data to the NN. The flexibility of the NN allows many configurations for introducing the descriptors into the model. Consider *d*_*m*,*n*_ an element of the input tensor ***D***, where *n* is the descriptor property (Δϵ_*ia*_, , or *R*_*ia*_), and *m* is the considered *a* ← *i* monoexcitatiton label. We used two strategies to introduce our data into the neural network: (1) the **1D model**, where each sample *j* is described by an array of 3*m* elements (one-dimensional), where all properties are introduced sequentially: , and (2) the **2D model**, where the descriptor properties for each *m* monoexcitation are grouped forming a two-dimensional tensor of (*m*,3) dimension, since all these properties are required to describe a particular excitation: .

The use of different properties, units, and ranges of magnitude may affect the learning process. It is always recommended to perform data preprocessing in order to give the same weight for all properties and hence to ease the learning process. In this work, we decided to scale all the data between [0, 1] using the tool *MinMaxScaler* provided by the *preprocessing* package of *scikit-learn*.^54^ Since the range of transition dipole moments is always positive and presents a high density distribution for values between 0 and 1, we transformed this property to a logarithmic scale.

**Figure 2:**
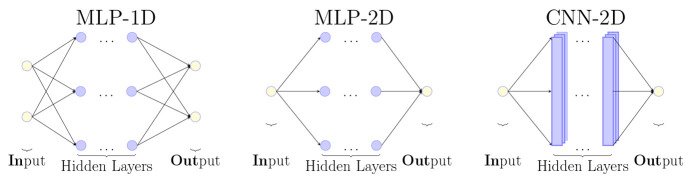
Simplified view of the topology built on top of *Keras*(55) and *Tensorflow*.^56^ From left to right, MLP-1D, where the input data is treated independently, and MLP-2D and CNN-2D, where the input data is treated as a combination of descriptors in both cases. See text.

Alongside with the preprocessing methods several NN models have been tested to find which will best perform for predicting properties. Figure 2 represents the different ML models tested in this work.

We construct our models using Keras and TensorFlow^55,56^ with the hyperparameters shown in Table 1 resulting from a Bayesian Optimization against the accuracy values obtained over the test set. Since the number of hyperparameters is large, only two have been optimized: 1) the number of layers and 2) the number of epochs. The activation function, which transforms the values between neurons across layers, has been selected empirically resulting from the use of the eLU^57^ and ReLU^58^ functions. Both activation functions were alternated starting with eLU. The full description of the models used in this work can be found in the Supporting Information.

**Table 1:** Relevant Hyperparameters Used to Build Our Models^a^

model	epochs	n. hidden layers	activation function
MLP-1D	1756	17	eLU/ReLU(negative slope = 0.01)^57,58^
MLP-2D	1419	4	eLU/ReLU(negative slope = 0.01)^57,58^
CNN-2D	1500	2	eLU/ReLU

a The second and third columns show the number of *Epochs* and *Hidden Layers*, respectively, as optimized for the model shown in the first column. The fourth column specifies the type of activation function and properties. For a complete list, see the Supporting Information.

By using this configuration, the training process with 10k molecules as a learning set and the prediction of 1k molecules from the test set takes  s for the slowest NN (CNN-2D model) on a commercial laptop with an Intel i7 and 12 Gb of RAM.

Table 2 shows the required times for the training and predicting processes. It is important here to remark that once the model is trained, the prediction of the spectroscopic properties is almost instantaneously obtained. Comparing this performance with the arduous task of solving the complex TDDFT equations shows the potential of using such an ML tool.

**Table 2:** Approximate *Training* and *Predicting* Times in Seconds (s) for Each Model Using 10k Molecules and 1k Molecules, Respectively^a^

model name	use *log*	training process (s)	predicting process (s)	total time (s)
MLP-1D	yes	919	<1	919
MLP-2D	yes	319	<1	319
CNN-2D	yes	1521	<1	1522

a Even if CNN-2D takes 1521 s to train, which is the slowest one, the response when predicting the properties is almost instantaneous.

The learning process is obviously biased by the learning data set. In order to validate the input data distribution, the optimized model, and its uncertainty, Musi et al. proposed to systematically perform a stability test.^59^ The learning data set is validated by repeating the neural network construction process (training and predicting process) over 10 experiments, by randomly selecting 10k learning molecules out of 15k PBE0 available calculations. We used 1k of the remaining 3k molecules as a control, which will remain unchanged across all experiments.

**Figure 3:**
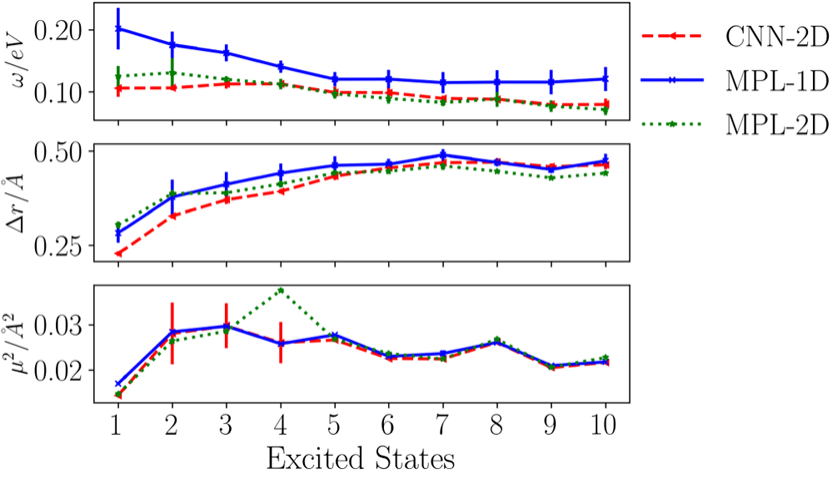
Mean absolute error (MAE) on the prediction of the excited-state energies Ω (top), charge-transfer character index Δ***r*** (middle), and transition moments μ^2^ (down) for 10 low-lying states averaged over the 10 repetitions of the same ML model. The MAE standard deviation of those experiments is also represented by the bar amplitude.

Figure 3 shows the mean average errors (MAEs) for the first 10 excited states of 1k 8CONF small molecules used in the control set. For each state, the mean value of the error over the 10 experiments is represented, and its standard deviation is depicted as a bar line.

We can see that CNN-2D and MPL-1D models reach errors of the excitation energy predictions that are close to chemical accuracy ( eV). Besides, they can also correctly predict the charge-transfer character of the low-lying excited state. Notice that Figure 1 shows Δ***r*** property as ranging from 0 to 4 Å. It means that a resulting error being smaller than 0.50 Å clearly distinguishes between short- and long-range charge-transfer characters. Regarding the transition moment prediction, we see that the models can just fairly predict the first excitation probability.

In the following section, we discuss the spectroscopic properties obtained using the more promising models, and we will remark on their strength and drawbacks.

## 2 Results and Discussion

As already described above, the main goal of this work is to find an adequate molecular-electronic descriptor that enables us to obtain accurate spectroscopic properties using machine learning techniques. From the analysis of accuracy and stability (Figure 3), we see that CNN-2D and MLP-2D produce the lowest error for predictions. Therefore, we selected these models to perform a deeper analysis.

**Figure 4:**
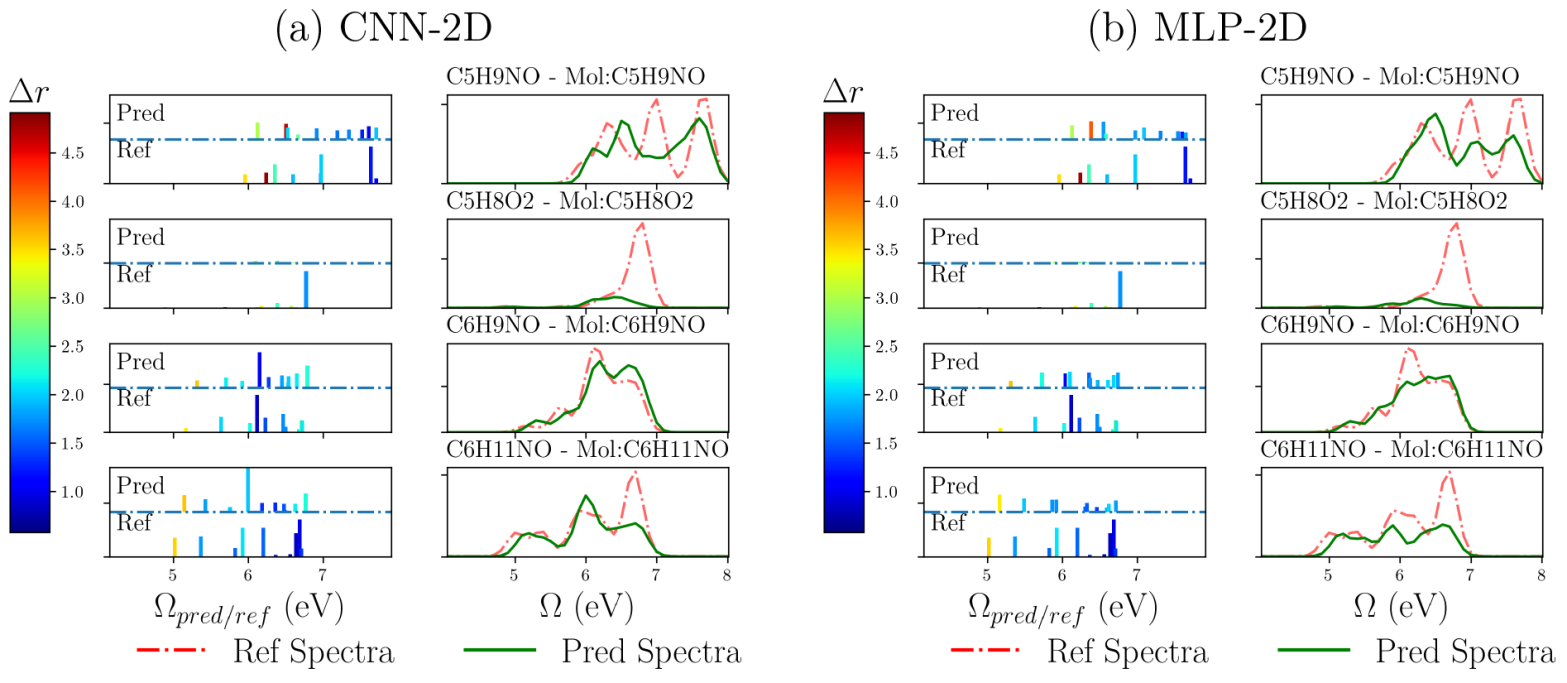
Discrete and broadened excitation spectra obtained with CNN-2D (a) and MLP-2D (b) for some example molecules contained in the control group. On the *X* axis are the calculated excited-state energies Ω, and on the *Y* axis appear their oscillator strengths, *f*_*I*_. Green curves represent reconstructed spectra from NN predictions, while the red ones represent those from PBE0-CASIDA calculations.

In order to easily visualize the agreement of the models for predicting the optical response of a molecule, we must also look at the absorption spectra. Figure 4 shows some examples of reconstruction for discrete and broadened absorption spectra (More examples can be found in the Supporting Information.).

The discrete spectra is represented as impulses positioned at the specific excitation energy of a given state. Their heights are proportional to the calculated oscillator strength. The shown color of each impulse represents the index for charge-transfer character according to the color scales of Δ***r*** on the left side. It is well-known that LDA functionals tend to underestimate excitation energies between Kohn–Sham’s orbitals when they involve a charge-transfer process. This is avoided in TDDFT calculations because of the consideration of a fraction of the exact Hartree exchange potential for hybrid functionals, such as PBE0. If we look at the spectra of C_5_H_9_NO, the first excited-state energy and nature in Figure 1, that was obtained with simpler LDA calculation results used for NN learning, they can be compared with the predicted results of Figure 4. A switch on the charge-transfer character of the first excited state appears together with a blue shift of the energy in the prediction. It means that even though our models were fed with rough LDA calculated properties, the learning process appears to add the effect of the exchange-correlation, being this is one of the more time demanding parts when computing spectroscopic properties by TDDFT routines. It could be very significant to save computational resources and time for excited-state predictions of large molecules.

Besides, the broadened spectra shown in Figure 4 have been reconstructed as a sum of Gaussians centered at the excitation energy which area is proportional to the oscillator strength. The broadening factor has been chosen to be 0.15 eV at the half width at the half-maximum (HWHM). We used the cross-correlation between the normalized spectra, the curve shift, and the curve area difference for comparison metrics between reference and predicted broadened spectra, because the relevant property in spectroscopy is usually the relative intensity instead of its absolute value. Our models sometimes have difficulties in distinguishing between closely lying excitations and can produce a switch between states. However, the broadening procedure enables us to mitigate this error by producing a good estimation of the continuum absorption spectra.

**Figure 5:**
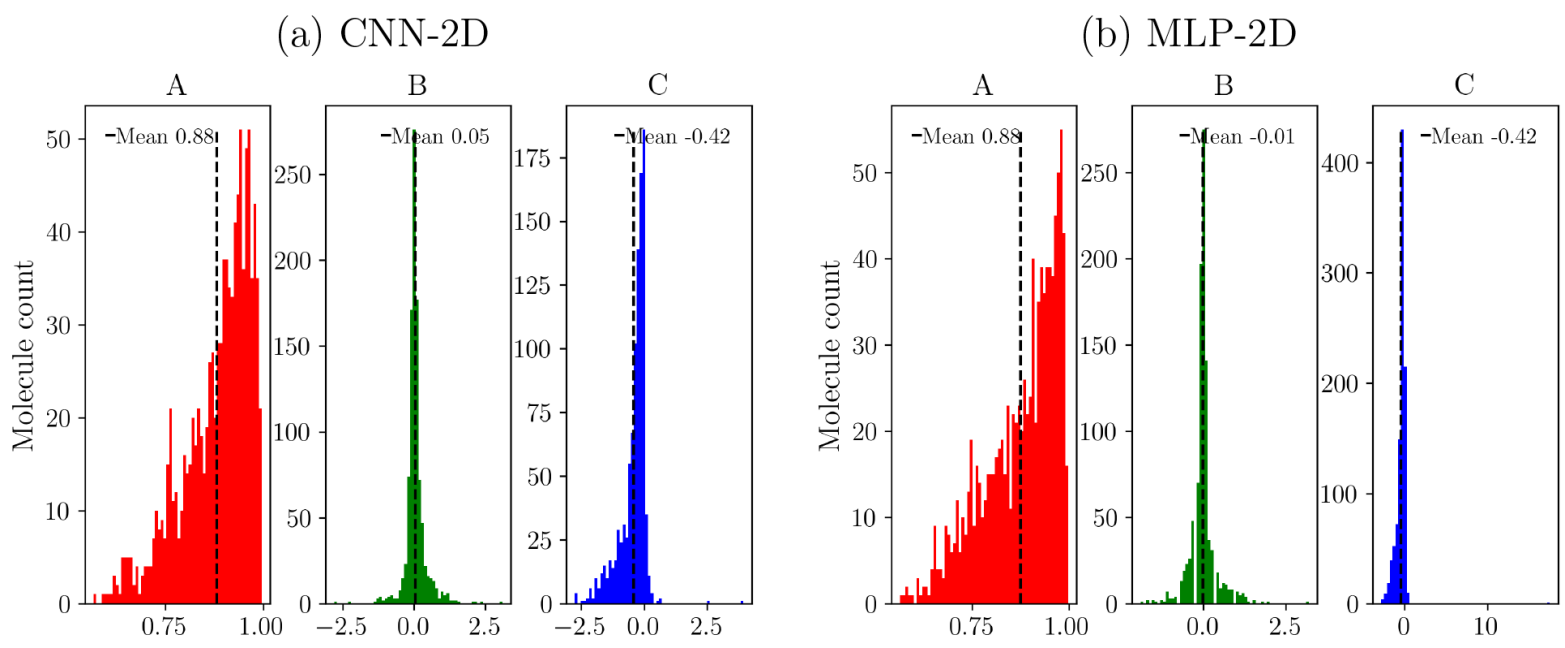
Distribution of the different metrics used to evaluate the prediction of the broadened spectra: (A, red) cross-correlation, (B, green) area under the curve, and (C, blue) curve shift for (a) CNN-2D and (b) MLP-2D.

Figure 5 shows the distribution of the parameters used to evaluate the obtained broadened spectra. Both models show a very good distribution of the cross-correlation values having more than 93% situated above a value of 0.90. In addition, almost all tests produce an area under the curve close to the 0 values, which indicates that the number of electrons is conserved in the prediction. Regarding the shift of the predicted broadened spectra, we observe that in spite of the fact the great majority presents just a slight shift, both models tend to produce a small red-shift of the absorption spectra when comparing with the PBE0-CASIDA results.

**Figure 6:**
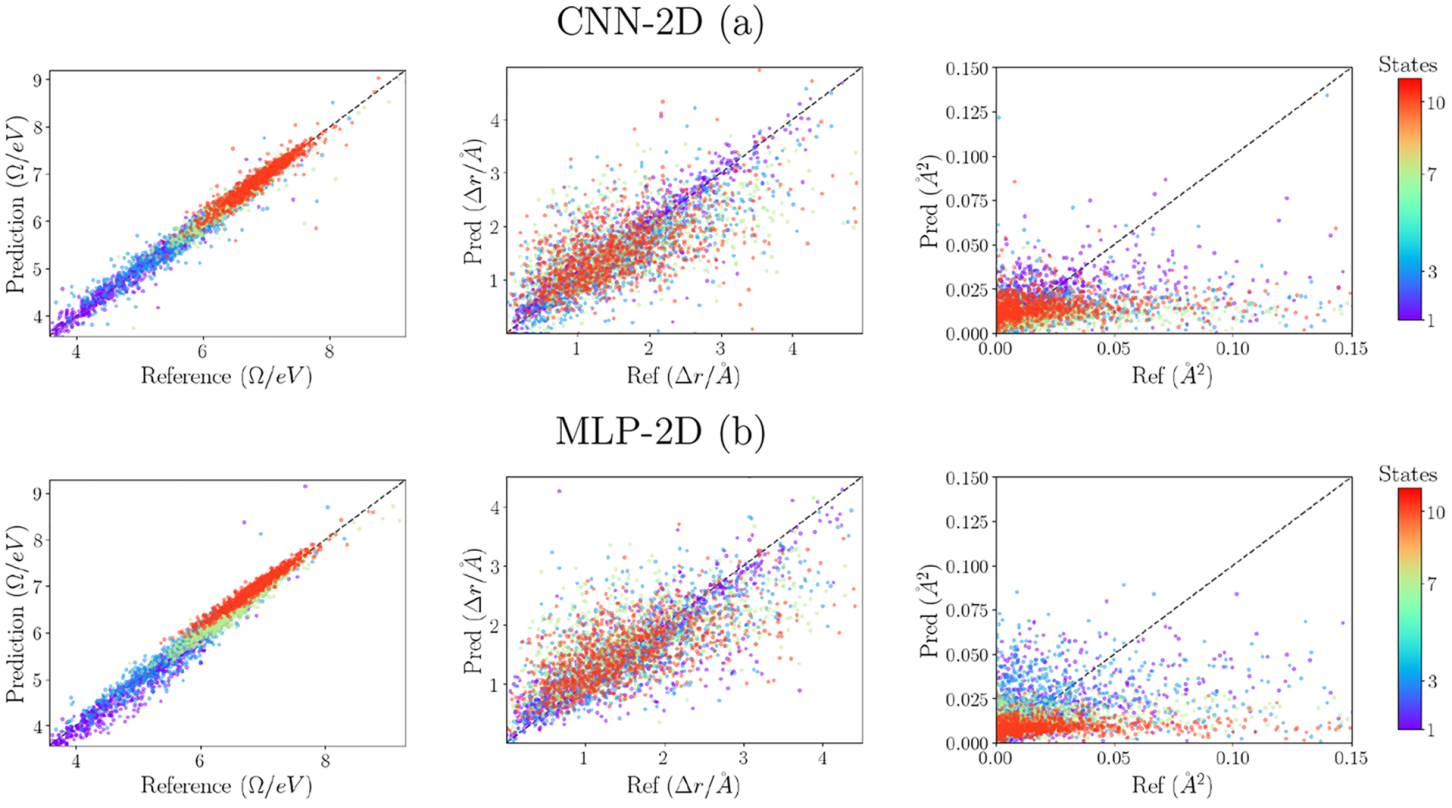
Correlation graphics between predicted and calculated values for best models (CNN-2D (a) and MLP-2D (b)). From left to right, the following are represented: excitation energies (Ω), charge-transfer coefficients (Δ*r*), and transition moments (μ^2^). The color of the points corresponds to the state number as ordered from the lowest energy according to the scale on the right-hand side.

Figure 6 shows the correlation graphics between the predicted values and the reference for each of the spectroscopic properties analyzed in this work. We can see that our models produce a very good correlation of the excitation energies for all excited states. A better correlation is observed for higher excitation energies, which can be attributed to a higher density of states found in the database at such frequencies. Regarding the charge-transfer index, we see that the CNN-2D model produces better correlation for the low-lying states, while it loses this correlation for higher states. It seems that both models hardly reproduce the proper transition dipole moments, being the CNN-2D model is slightly better for the low-lying states. A possible source of error can be attributed to the diverse distribution of the values that increases the complexity of the learning process and/or to intrinsic inconsistencies of the theoretical calculations of transition dipole moments when Kohn–Sham’s virtual orbitals are involved. Other tests performed by increasing the size of our learning set including up to 15k molecules suggest that enlarging the database can improve the correlation of the Δ***r*** for higher excitations but just produces a slight improvement in the transition dipole moments.

## 3 Conclusions

Accurate knowledge of the spectroscopic properties of molecules has been of great interest since long ago for academical as well as industrial sectors. The high computational cost of the quantum chemistry/physics techniques, mostly for large molecules, and the lack of an extended experimental database, as well as the increase on the reliability of the machine learning and artificial intelligence methods, are inviting researchers to apply those techniques for predicting such physical properties. Nevertheless, the major difficulty usually relies on finding the proper descriptors being able to correlate with properties of interest.

As mentioned above, the main objective of this work is to find adequate molecular-electronic descriptors to be used with proper NN models to predict the theoretical absorption spectra for a group of small organic molecules. We prove that the combination of certain selected electronic properties (Δϵ_*ia*_, *R*_*ia*_, ) resulting from low cost *ground state LDA* calculations appears as good descriptors for the prediction of such spectroscopic properties at a higher level of theory (e.g., TDDFT with the PBE0 functional without a geometry relaxation). Besides, we demonstrate that a simple optimized Convolutional Neural Network (CNN-2D) as well as a Multi Layer Perceptron (MLP-2D) network can learn to supply the exchange correlation correction required for predictions going from LDA to PBE0 levels of theory.

Many others advantages arise as (i) the trained NN model can be reused for further predictions on previously unknown molecules; (ii) geometry optimizations are not required although could be recommended; and (iii) the trained NN model expects as input the ground state LDA electronic data, and it can be obtained from any (TD)DFT code that managed to produce this output.

Previous works were focused on the prediction of the first excited state^1^ or on the density of states near to the LUMO^2^ by using only geometrical or spacial descriptors. In this work, we demonstrate the need of an electronic descriptor not only to extend the prediction of the excitation energies at chemical accuracy but also to give information about their charge-transfer character. Oscillator strength values proved to be the most challenging property for our models. Although we demonstrate an enhancement on the prediction of the low-lying excitation probabilities when the training set is augmented, the transition dipole moments for high energy excitation remained poorly correlated. Different sources of error that can be addressed for this problem are discussed.

We can conclude that by constructing a large database which would include all types of molecules (small, medium, and large sized molecules), the presented method would be able to precisely obtain spectroscopic properties for any unknown molecule by just computing a low-cost DFT ground state using the LDA functional. This goal can be achieved by using, for example, the Novel Materials Discovery Laboratory (NOMAD) open database.^60^

The natural next step is to provide even more fundamental properties as descriptors to the Neural Network. However, in progress work reveals that the use of the same properties coming from unoptimized Linear Combination of Atomic Orbitals (LCAO) calculations, typically used to begin a ground state calculation, requires a more complex network optimization to overcome the big gap between an LCAO level of theory and that of PBE0 hybrid functional calculations. Besides, we are also working on the use of the simplest ML techniques to predict optimized geometries, that could avoid the tedious task of geometry relaxations.^61^

All data from the calculations done in this work have been stored in the database of the Novel Material Discovery Laboratory (NOMAD) project^60^ and can be downloaded: LDA from 10.17172/NOMAD/2021.10.18-2 and PBE0 from 10.17172/NOMAD/2021.10.18-3.

## Supporting Information Available

Additional data and NN descriptions (PDF)
