# Supplementary material for: Electronic Descriptors for Supervised Spectroscopic Predictions
Source: J Chem Theory Comput. 2023 Mar 6;19(6):1818–26. doi: 10.1021/acs.jctc.2c01039 (PMC10061681; doi:10.1021/acs.jctc.2c01039)
Supplement: Supplementary file 1 — ct2c01039_si_001.pdf [file ct2c01039_si_001.pdf]

# Supplementary Information: Electronic Descriptor for a Supervised Spectroscopic Prediction

Carlos Manuel de Armas Morejón,[*,†] Luis Montero Cabrera,[*,†] Angel Rubio,[*,‡] and Joaquim Jornet-Somoza[*,¶,‡]

†*Laboratorio de Química Computacional y Teórica, Facultad de Química, Universidad de La Habana, 10400. La Habana, Cuba.*

‡*Theory Department, Max Planck Institute for the Structure and Dynamics of Matter and Center for Free-Electron Laser Science, Luruper Chaussee 149, 22761 Hamburg, Germany*

¶*Nano-Bio Spectroscopy Group and ETSF Scientific Development Centre, Department of Materials Physics, University of the Basque Country, CFM CSIC-UPV/EHU-MPC and DIPC, Tolosa Hiribidea 72, E-20018 Donostia-San Sebastián*

E-mail: carlosdearmasm@gmail.com; lmc@fq.uh.cu; angel.rubio@mpsd.mpg.de; j.jornet.somoza@gmail.com

## 0.1 Description of the Bayesian Optimization

The number of hidden layers and epochs for each topology to be determine by Bayesian Optimization. For epochs we select the values $1, 100, 500$, $1000, 1500$ and for hidden layer the values $1, 2, 5, 10, 20, 30$. The combinations of this values form the search space for the Bayesian Optimization. Figure 1 shows the hyper-surface resulting of the evaluation of the models. The red spots are where the models gives the worst possible results and the blue

areas are where the combination of parameter gives the best possibles results. Those blue areas point to our selected combinations of hidden-layers and epochs. The metric used was the *Accuracy* implemented by Keras.

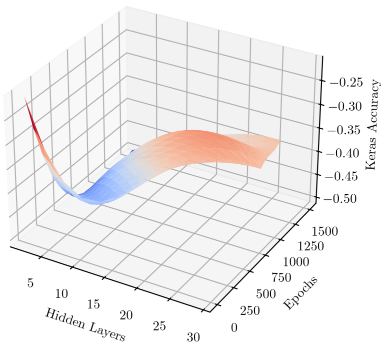


[CNN-2D]

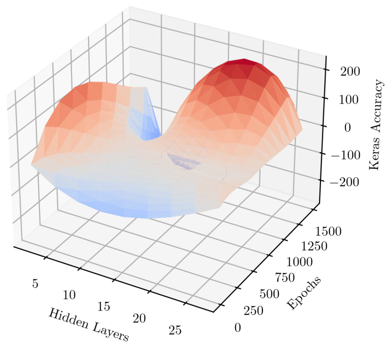


[MLP-1D]

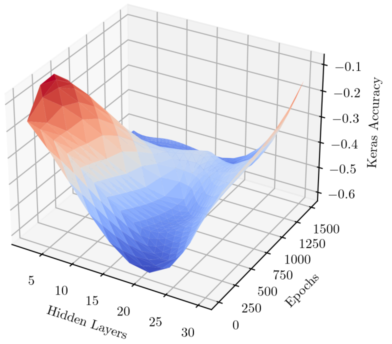


[MLP-2D]

Figure 1: Accuracy vs (Hidden-Layers, Epochs) of our models for the Bayesian optimization. Using LDA-GS as descriptor and PBE0-CASIDA as target property. In *red* those combination where the models gives the worst resultes, in *blue* the best combination of hyper-parameters.

Table 1 shows the rest of hyper-parameter values selected to construct out models. The model where constructed using Keras with TensorFlow, the names coincides with those implemented in by the frameworks named before.

Table 1: List of hyper-parameter for our models and how their values were determine.

| Model | Activation | Number of Neurons per Hidden-layer | Optimizer | Loss Function | Kernel size |
|---|---|---|---|---|---|
| MLP-1D | eLU/ReLU(negative slope = 0.01) | 60 | Adams | MSE | - |
| MLP-2D | eLU/ReLU(negative slope = 0.01) | 25 | Adams | MSE | - |
| CNN-2D | eLU/ReLU | 60 (Filter) | Adams | MSE | 20 |

## 0.2 Reconstruction of the discrete absorption spectra.

As an example of multiple molecules discrete absorption spectra, Figure 2 shows 20 molecules for CNN-2D and MLP-2D models. In both cases the function *log* was used as a pre-possessing method.

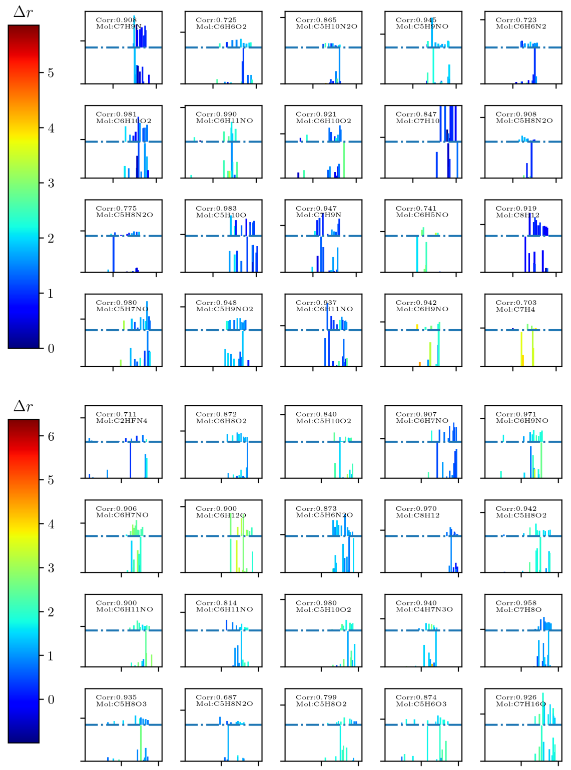

[CNN-2D] [MLP-2D]

Figure 2: Discrete absorption spectra reconstruction. The color scale shows the intensity of the transition measured by the $\Delta r$ metric. Each image shows a molecule, were the upper reconstruction is the prediction a the lower reconstruction is the reference. The descriptor used was extracted from LDA-GS and the target properties from PBE0-CASIDA.

For simplicity Figure 3 show the best, mean and worst spectra reconstruction.

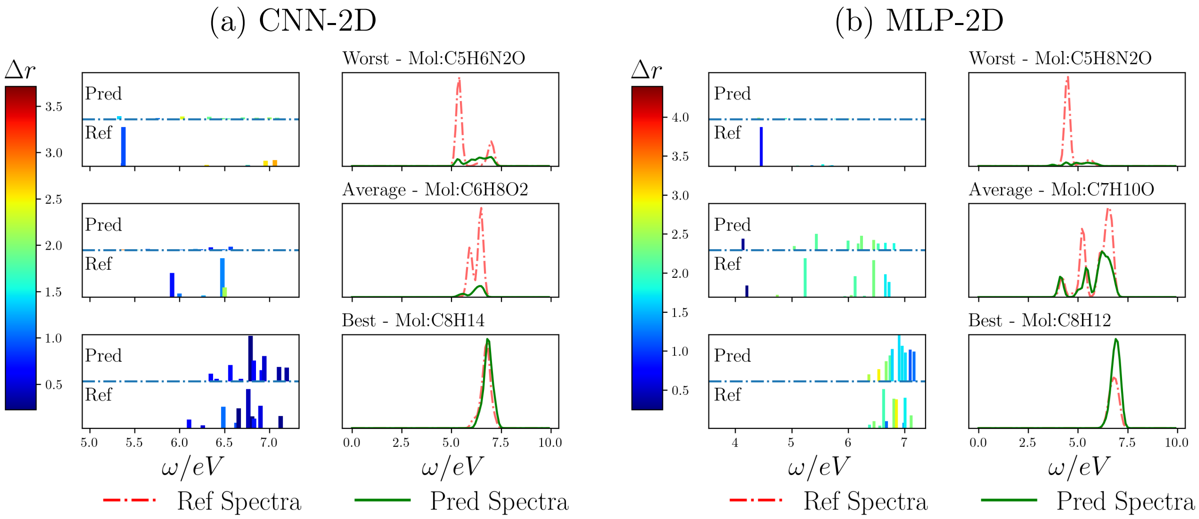


Figure 3: Discrete and broadened excitation spectra for the best, mean and worst examples: (a) CNN-2D (b) MLP-2D. On the $X$ axis are the excites states $\omega$ and on the $Y$ axis the oscillator strengths $f_I$. Green curves represent reconstructed spectra from predictions, while the red ones represent the reference reconstructed spectra from PBE0-CASIDA calculations.
